# Acute Pancreatitis among Patients Visiting the Department of Surgery in a Tertiary Care Centre: A Descriptive Cross-sectional Study

**DOI:** 10.31729/jnma.8014

**Published:** 2023-02-28

**Authors:** Sunil Basukala, Bishnu Deep Pathak, Pravakar Dawadi, Sujan Bohara, Ayush Tamang, Soumya Pahari, Yugant Khand, Ojas Thapa, Ujwal Bhusal

**Affiliations:** 1 Department of Surgery, Shree Birendra Hospital, Chhauni, Kathmandu, Nepal; 2 Nepalese Army Institute of Health Sciences, Sanobharyang, Kathmandu, Nepal

**Keywords:** *acute pancreatitis*, *gastrointestinal disease*, *prevalence*

## Abstract

**Introduction:**

Acute pancreatitis is the inflammation of pancreatic parenchyma characterised by severe abdominal pain and nausea. It is a common gastrointestinal disease requiring hospital admission. The death rate for mild acute pancreatitis is low but severe acute pancreatitis can reach up to 40%. This study aimed to find the prevalence of acute pancreatitis among patients attending the Department of Surgery in a tertiary care centre.

**Methods:**

This descriptive cross-sectional study was conducted from 1 October 2021 to 30 March 2022. The study was conducted after receiving ethical approval from the Institutional Review Committee (Registration number: 454). Patients with age more than 18 years were included and patients less than 18 years of age including those suffering from chronic pancreatitis, pancreatic malignancy or immunocompromised states were excluded. Convenience sampling was done. Point estimate and 95% Confidence Interval were calculated.

**Results:**

Among 1560 patients, the prevalence of acute pancreatitis in our study is 120 (7.69%) (2.9212.46, 95% Confidence Interval). Out of them, 57 (47.50%) were males and 63 (52.50%) were females. Out of total, hypertension found in 52 (43.33%) was the most common co-morbidity observed followed by diabetes mellitus 18 (15%). Similarly, 80 (66.67%) patients had mild pancreatitis whereas 40 (33.33%) had moderate pancreatitis and 8 (6.67%) had severe pancreatitis.

**Conclusions:**

The prevalence of acute pancreatitis among hospital admissions in the department of surgery in a tertiary care centre was found to be similar to other studies done in a similar setting.

## INTRODUCTION

Acute pancreatitis is the inflammation of pancreatic parenchyma characterised by severe abdominal pain and nausea.^1^ Alcohol and gallstones are the major causes accounting for as much as 95% of the cases.^2^

It is a common gastrointestinal disease requiring admission to the hospital, with an estimated global incidence of 33.74 cases per 100,000 person-years and 1.60 deaths per 100,000 person-years.^3^ Mild acute pancreatitis has a very low mortality rate of less than 1%,^4,5^ whereas the death rate for severe acute pancreatitis can be 20 to 40% depending on the development of organ failure and secondary infection of pancreatic and peripancreatic collections.^6,7^

The objective of this study was to find the prevalence of acute pancreatitis among patients attending the department of surgery in a tertiary care centre.

## METHODS

This descriptive cross-sectional study was conducted at Shree Birendra Hospital from 1 October 2021 to 30 March 2022 for the period of 6 months after receiving ethical clearance from the Institutional Review Committee of the Nepalese Army Institute of Health Sciences (Registration number: 454). All patients, aged 18 years or more admitted to the surgical ward of the hospital within the study period were included in the study. Patients of less than 18 years of age or suffering from chronic pancreatitis or pancreatic malignancy or with immunocompromised states were excluded from the study. Informed consent was obtained from all the patients. Convenience sampling method was employed.

The sample size was calculated using the following formula:


$$\begin{array}{ll} \text{n} & ={\text{Z}}^{2}\times \frac{\text{p}\times \text{q}}{{\text{e}}^{\text{2}}}\\ & =1.96^{2}\times \frac{0.0617\times 0.9383}{0.02^{2}}\\ & =556 \end{array}$$

Where,

n = minimum required sample size
Z = 1.96 at 95% Confidence Interval (CI)
p = prevalence as reported by a previous study, 6.17%^8^
q = 1-p
e = margin of error, 2%

Adding a 10% non-response rate, the required sample size becomes 618. On doubling, the sample size becomes 1236. However, we took a total of 1560 patients.

Data was collected through a self-administered questionnaire consisting of patient demographics, BMI, associated co-morbidity, clinical features at the time of presentation, cause of pancreatitis and severity of pancreatitis according to the revised Atalanta classification.^9^ All the patients were sent for transabdominal ultrasonography. Similarly, in all the study participants, serum amylase, serum lipase, complete blood count (CBC), serum electrolytes, renal function tests (RFTs), liver function tests (LFTs), and serum calcium, blood glucose, and c-reactive protein (CRP) levels were tested. Furthermore, their systemic complications (if any), surgical interventions (if done), length of hospital stay and their outcome were noted from the patient's case file. All the patients were described the purpose of the study and informed consent was taken before including them in the study.

Data were analyzed using the IBM SPSS Statistics version 22.0. Point estimate and 95% CI were calculated.

## RESULTS

Among 1560 patients, the prevalence of acute pancreatitis in our study was 120 (7.69%) (2.92-12.46, 95% CI) in the Department of Surgery. The median age of the patients having acute pancreatitis was 42 (Range: 32-47) years. Out of them, 57 (47.50%) were males and 63 (52.50%) were females. The average body mass index (BMI) was 25.70 kg/m^2^ (Range: 23.40-27.10). Hypertension 52 (43.33%) was the most common co-morbidity followed by diabetes mellitus 18 (15%). A total of 67 (55.83%) cases had a history of chronic smoking. Similarly, 80 (66.67%) patients had mild pancreatitis whereas 40 (33.33%) had moderate pancreatitis and 8 (6.67%) had severe pancreatitis.

**Figure 1:**
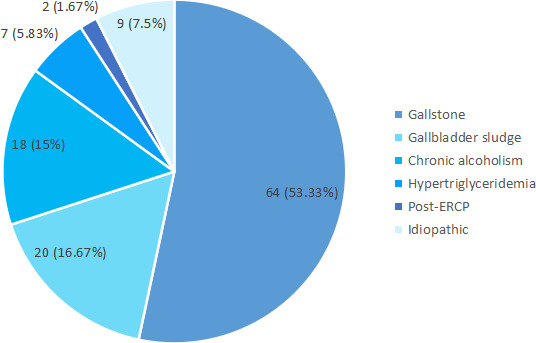
Pie chart showing underlying conditions observed among patients with acute pancreatitis (n= 120).

The most common underlying condition observed among patients having acute pancreatitis was gallstone 64 (53.33%) followed by gallbladder sludge 20 (16.67%) and chronic alcoholism 18 (15%). Biliary factors include gallstone and gallbladder sludge. Hence, biliary factors were observed in 84 (70%) of the total cases of acute pancreatitis (Figure 1).

Out of total acute pancreatitis cases, 102 (85%) cases presented with nausea and vomiting. A total of 7 (5.83%) cases had jaundice at presentation out of which 5 (71.43%) cases had moderate to severe pancreatitis (Table 1).

**Table 1:** Clinical presentation and laboratory indicators among acute pancreatitis patients (n= 120).

Variables	Total	Mild	Moderate to Severe
Clinical features at presentation	n (%)	n (%)	n (%)
Nausea and vomiting	102 (100)	65 (63.73)	37 (36.27)
Jaundice	7 (100)	2 (28.57)	5 (71.43)
Laboratory indicators			
Serum amylase (U/L)			
< 110	7 (100)	6 (85.71)	1 (14.29)
> 110	53 (100)	44 (83.02)	9 (16.98)
> Three times	60 (100)	30 (50)	30 (50)
Serum Lipase(U/L)			
< 300	2 (100)	1 (50)	1 (50)
> 300	74 (100)	58 (78.38)	16 (21.62)
>Three times	44 (100)	21 (47.73)	23 (52.27)
TLC (per cubic mm)			
< 11,000	86 (100)	57 (66.28)	29 (33.72)
> 11,000	34 (100)	23 (67.65)	11 (32.35)
Serum Calcium(mg/dl)			
< 8.5	10 (100)	1 (10)	9 (90)
> 8.5	110 (100)	79 (71.82)	31 (28.18)
Blood Glucose(mg/dl)			
< 100	17 (100)	13 (76.47)	4 (23.53)
> 100	103 (100)	67 (65.05)	36 (34.95)
Serum Bilirubin(mg/dl)			
< 1.2	102 (100)	69 (67.65)	33 (32.35)
> 1.2	18 (100)	11 (61.11)	7 (38.89)
CRP (mg/dl)			
< 150	79 (100)	58 (73.42)	21 (26.58)
> 150	41 (100)	22 (53.66)	19 (46.34)
Serum Creatinine(mg/dl)			
Raised	21 (100)	11 (52.38)	10 (47.62)
Normal	99 (100)	69 (69.70)	30 (30.30)
USG abdomen			
Bulky pancreas	99 (100)	66 (66.67)	33 (33.33)
Normal pancreas	21 (100)	14 (66.67)	7 (33.33)

The mortality was observed in 1 (0.83%) patient. The median duration of hospital stay was 4 (3-5) days. Out of all cases of acute pancreatitis, only 1 (0.83%) needed surgical interventions.

## DISCUSSION

Acute pancreatitis is an acute inflammatory process of the pancreas. The revised Atlanta classification requires that two or more of the following criteria be met for the diagnosis of acute pancreatitis: (a) abdominal pain suggestive of pancreatitis, (b) serum amylase or lipase level greater than three times the upper normal value, or (c) characteristic imaging findings.^9^ Our study identified the prevalence of acute pancreatitis among hospital admissions in the department of surgery as 7.69%.

The median age group of the patients having acute pancreatitis in our study was 42 (32-47) years. Studies suggest that the age and sex distribution of acute pancreatitis vary according to the aetiology.^10,11^

Furthermore, the risk of developing acute pancreatitis rises with increasing age.^12^ In our study, among the patients having acute pancreatitis, 52.50% and 47.50% were women and men respectively. On contrary to that, most studies suggest that men have a higher preponderance than women to acute pancreatitis.^11-13^

The most common presentation of acute pancreatitis in women includes gallstones, endoscopic retrograde cholangiopancreatography or autoimmune diseases, or idiopathic.^12^ In our study, 53.33% of patients having acute pancreatitis presented with gallstones. Similar kinds of findings can be observed in other studies as well.^14^ Apart from biliary factors like gallstone (53.33%) and gallbladder sludge (16.67%), the most common non-biliary factor observed was chronic alcoholism (15%) among patients having acute pancreatitis. Studies have shown a link between acute pancreatitis and the intake of alcohol.^15,16^ A systematic review suggests that tobacco smoking increases the risk of acute and chronic pancreatitis.^17^ In accordance with that, most of the patients with acute pancreatitis (55.83%) in our study had a history of smoking.

There are limited studies on the prognosis of concomitant hypertension and acute pancreatitis. However, a study suggests that the presence of arterial hypertension can have a pronounced effect on pain in a patient with chronic pancreatitis.^18^ In our study 43.33% had hypertension at the time of diagnosis of acute pancreatitis. From various studies, there has been adequate evidence suggesting the development of diabetes mellitus following acute pancreatitis.^19-21^ There is a risk of developing acute pancreatitis due to the structural changes and hyperglycemic crisis occurring as a result of diabetes mellitus.^22,23^ About 15% of the patients in our study had concomitant diabetes mellitus. Nevertheless, the underlying conditions observed among patients with acute pancreatitis were either biliary factors or alcohol. None of the subjects in our study solely had diabetes mellitus and acute pancreatitis.

In our study 85% of the patients with acute pancreatitis presented with nausea and vomiting. Similarly, 83.9%^24^ and 90% of patients presented with nausea and vomiting respectively in other studies.^25^ Our study showed the three-fold rise in serum amylase and serum lipase to be present in 50% and 36.67% of the patients admitted with acute pancreatitis respectively. A similar trend can be observed in other studies where a three-fold rise in serum amylase was observed in 47%^26^ while a three-fold rise in serum lipase observed in comparatively more patients, i.e. 82%.^27^

Likewise, our study showed leukocytosis (TLC>11,000/ mm^3^) in 23.33% of the patients with acute pancreatitis, which is lower than the findings from the studies which showed leukocytosis in two-thirds of the patients.^24,26^ Previous studies have reported decreased serum calcium among 8.6%^25^ and 18%^26^ of the patients with acute pancreatitis. The findings from these studies were similar to our study where 8.3% of the patients having acute pancreatitis presented with hypocalcemia (<8.5 mEq/L).

CRP at admission was raised in 34.1% of total cases of acute pancreatitis in our study. A previous study shows patients who died from acute pancreatitis had high CRP values on admission (>160 mg/dl).^27^ In contrast to that, another study showed no link between raised CRP and mortality.^24^ CRP levels at the time of admission have been found to predict severity poorly, however, CRP >150 mg/dL within the first 48 hours has shown high sensitivity for predicting the severity of acute pancreatitis.^28,29^

In our study, 69.7% of patients with normal serum creatinine had mild acute pancreatitis. A previous study showed that raised serum creatinine at admission is not associated with an increase in the incidence of pancreatic and extra-pancreatic complications.^26^ Similarly, another study showed that normal serum creatinine had a high negative predictive value for necrotizing pancreatitis.^30^ The mortality rate among patients with acute pancreatitis in our study was 0.83% which is relatively lower than another study where mortality was observed in 5% of patients with acute pancreatitis,^25^ Whereas, similar findings to our study can be observed in other studies where 2% of mortality was observed.^26^

Our study was based on data from one tertiary care centre. So, it cannot be generalized to the broader prospect. But since this centre is the only referral centre for the Nepalese Army, this data provides a close representation of the Nepalese Army family.

## CONCLUSIONS

The prevalence of acute pancreatitis among hospital admissions in the department of surgery in our centre was found to be similar to other studies in a similar setting. Biliary factors followed by alcohol intake were observed to be the most common underlying condition observed among patients with acute pancreatitis.
